# Institutionalisation Is a Vital Element for Fairness of Priority Setting in the Package Design if the Target is Universal Health Coverage: Comment on "Evidence-Informed Deliberative Processes for Health Benefits Package Design – Part II: A Practical Guide"

**DOI:** 10.34172/ijhpm.2022.7544

**Published:** 2022-12-12

**Authors:** Haniye Sadat Sajadi, Mohamed Jama, Reza Majdzadeh

**Affiliations:** ^1^Knowledge Utilization Research Center, University Research and Development Center, Tehran University of Medical Sciences, Tehran, Iran.; ^2^Ministry of Health, Federal Government of Somalia, Mogadishu, Somalia.; ^3^Interdisciplinary Research and Practice Division, School of Health and Social Care, University of Essex, Colchester, UK.

**Keywords:** Universal Health Coverage, Health Sector Reform, Essential Health Services, Priority Setting, Sustainability, Institutionalisation

## Abstract

The evidence-informed deliberative processes (EDPs) guide provides a practical framework for fair priority setting of the health benefits package (HBP) that countries can reasonably use. The steps presented in the EDPs are applicable for prioritising health services in designing HBP and are consistent with practical experience in countries. However, institutionalisation must be considered an element of fairness in the priority-setting process if the aim is to reach broader goals of a health system, such as universal health coverage (UHC). Otherwise, the EDPs for priority setting might not be integrated into the formal health system or impactful, resulting in a waste of time and resources, which is unfair. Institutionalisation means formalising the desired change as an embedded and integrated system so that the change lasts over time. For the institutionalisation of EPDs, four stages are suggested, which are (1) establishing a supportive legal framework, (2) designating governance and institutional structure, (3) stipulating the EDPs processes and (4) individual and institutional capacity building.

 Designing a health benefits package (HBP) is a policy choice and strategic action to move toward universal health coverage (UHC) and achieve Sustainable Development Goals.^1^ Several resources have been published to support the HBP design, including a list of cost-effective interventions by Diseases Control Priorities 3 (DCP3)^2^ (a new edition coming soon as DCP4) and the World Health Organization’s (WHO’s) UHC Compendium^3^ which is a web-based comprehensive list of services for each condition. Also, eight principles defined the design of HBP as follows: impartial for universality, democratic and inclusive with public involvement, based on national values and clearly defined criteria, data-driven and evidence-based, respect the difference between data, dialogue and decision, linked to robust financing mechanisms, include effective service delivery mechanisms, open and transparent in all steps.^4^ In addition, the “What’s in, what’s out”^5^ by the Center for Global Development and the WHO’s “Making Fair Choices report”^6^ introduced the concepts needed for the design of HBP. Nevertheless, a gap existed for a practical guide that countries could use in daily practices. The Oortwijn et al^7^ article and the guide prepared in parallel at Radboud University Medical Center by the same authors^8^ are a valuable response to this need that provide practical steps for HBP design through evidence-informed deliberative processes (EDPs). But the current commentary focuses on applying EDPs more broadly to health system strengthening and achieving UHC.

 The guide’s pertinence was assessed recently during the DCP3 country translation review of six countries involved in the design of HBP. This exercise reviewed the experiences of Afghanistan, Ethiopia, Pakistan, Somalia, Sudan, and Zanzibar (a semi-autonomous region in Tanzania).^9^ The findings illustrated that the process of EDPs introduced in the article and the guide could adequately cover the steps taken to design the HBP in these countries. All six experiences undertook stepwise activities to develop HBP akin to the EDPs guide. So, these steps are apt for the fair design of the HBP in these countries’ particular conditions and can be practically used to implement the elements of stakeholder involvement, evidence-informed evaluation, transparency, and appeal that are included in the ethical framework of Accountability for Reasonableness. Although health technology assessment (HTA) as an approach is being used for HBP development (in 65% of countries and areas according to the WHO global survey)^10^ and includes technical and procedural aspects of HBP development, HTA and HBP development are not necessarily synonymous. There is a critical difference between the countries dealing with HTA and the design of HBP, which is related to the context of their health system. In HTA, the approach in most cases is incremental, meaning an HBP exists, and this HBP is subject to optimisation. Usually, the health system in this situation is well established. However, the HBP design is in concurrence with overall changes or transitions of health systems in the country.

 Nowadays, many low- and middle-income countries undertake comprehensive HBP design as part of their reform to strengthen the health system and move towards UHC. The intention is not only to have a list of services but to use HBP as a base for health system strengthening.^11^ Among the six countries mentioned above, the design and implementation of the HBP was the operationalisation of a more general agenda of UHC. Therefore, the HBP design must be considered an action in the context of a more comprehensive movement of the health system reform, which is long-term and is not limited to a time-bounded project.

 A package should be designed, implemented, and revised from time to time whenever health needs change, more resources are mobilised and or new interventions are introduced. For countries with health systems in transition, a fair process is complemented if it is sustainable and results in a nationally owned and institutionalised priority-setting system.

 Institutionalisation means formalising the desired change as an embedded and integrated system so that the change lasts over time and has the necessary power to prevail.^12^ Therefore, apart from all the methodological details of this process, all legal, political, financial and system requirements must be provided for the EDPs institutionalisation.

 Institutionalisation is introduced in the guide as a requirement for EDPs’ success and an indicator for impact evaluation based on the theory of change. But the guide’s primary focus was the process (as is clear from the title). Institutionalisation was not presented explicitly in the guide, while its components have been introduced. The guide’s aim was general and not dealing with HTA or HBP as a part of the countries’ pathway to realise UHC (which was not the guide’s aim).

 If a country does not institutionalise this process, it will face challenges because it would be:

1. Temporary, which might not be a priority after a political change.
2. Unsuccessful and cause waste of resources.
3. Suffer from weak stakeholders’ participation and support.
4. Fail in contributing to the evidence ecosystem for policy-making.^13^

To strengthen the health system through EDPs, institutionalisation should be started from the beginning of the process.

 Now, if considering fairness as needs perceived by stakeholders, institutionalisation, as an effort for sustainability, should be added to the previous elements of fairness of the process in EDPs.

 Nevertheless, approaches to institutionalising EDPs can differ depending on the country’s context. Still, there are guides for the institutionalisation of HTA and evidence-informed policy-making as following which can be helpful^11^:

 (1) Establishing a supportive legal framework: all laws, regulations, executive orders, and endorsed guidelines related to actions for deciding which health services are eligible to be covered by the public fund should be identified. The recent WHO global survey of HTA found that more than half of countries have legislative requirements for HTA, yet it is legally binding in one-third of countries.^10^ Of course, this survey was self-explanatory and might have some overestimations. However, the gap between having legislative requirements and being legally bound is still pertinent and establishing a supportive proper legal framework is needed in many countries.

 It should determine what legal gaps exist to revise or formulate new ones. The legal hierarchy of the country should be considered if any changes are planned. It means any explicit requirement or guidance needs supportive executive order and must be aligned with upstream laws.

 An essential part of the institutionalisation of EDPs is the existence of a policy document, preferably endorsed at the level of parliament or at a level that makes it mandatory for the planning and finance sectors. This policy document must clearly state that financing each service from public resources is conditional on the EDPs and shows how to deal with deviation from this requirement. In addition, such a document specifies a health system strategy and reveals a solid political commitment to institutionalising EDPs.

 (2) Designation of governance and institutional structure: considering that different stakeholders are playing roles in the process of EDPs, a coordinating body is needed. Recognizing the different types of institutional arrangements would therefore be critical. The best approach is to use the capacity of existing institutions and not create a new structure. However, the roles and responsibilities of each player must be well-defined. The responsible body for each of the EDPs steps should be clear, and each must be accountable for their mandates. The experience of different countries in institutionalising the package definition for the realization of UHC indicates that stakeholders’ involvement, especially the community, plays a key role.^14^ Furthermore, the design and implementation of an HBP and UHC should become a public demand instead of a request from a limited number of experts. In other words, the political will and its sustainability are vital for the initiative’s sustainability and for moving towards UHC.^15^

 In this division of labour, avoiding conflict of interests is essential and must be considered in all work steps. Different stakeholders could have a variety of motivations. In some countries, the contribution of donors to the health system is highly prominent. Donors can have various agendas that might not necessarily align with the design of an HBP to realize UHC. Along with this, the private sector has a large share of the provision of health services in many countries. The crucial issue is how to set up the processes and enhance coordination to get the benefits of stakeholders’ participation, eg, donors and the private sector, while the conflict of interest is managed and country ownership is not compromised.

 (3) Stipulating the EDPs processes: it is necessary to make the process for conducting EDPs explicit and modify the current processes. For institutionalisation, it is required to approve and formalize all steps of the EDPs. In addition, these processes must align with other organisational modalities, such as accountability, and be embedded into the current processes. Therefore, the output of this step is having a description of all EDPs steps,^7^ including (*a*) installing a governance structure, (*b*) mapping and selecting services for evaluation, (*c*) defining decision criteria for prioritisation of services, (*d*) collecting evidence on decision criteria for services, (*e*) prioritising services, (*f*) developing implementation plan, (*g*) implementing communication and appeal, and (*h*) implementing monitoring and evaluation as standard operating procedure and endorsed by a relevant authority.

 (4) Individual and institutional capacity building: EDPs should be nationally owned. Human resources, infrastructure, information systems, and sustainable public financial resources are required. Knowing the status quo of human capital needed for priority setting is essential. In the early stages of conducting EDPs, the country may need more external technical assistance. Along with the country’s education system, new academic programs (such as HTA, health economics, epidemiology, health management, health policy, etc) should be established or, if existing, expanded. Staff development on leadership competencies, such as decision-making, critical evaluation and negotiation, is needed, similar to package design technical competencies. The role of individual and institutional capacity building is essential. Also, the leadership and/or the champions’ role is crucial to implement EDPs.

 An information system is the Achilles heel of the institutionalisation of any evidence-informed initiative. The status of the information and data system should also be evaluated as part of a monitoring and evaluation to determine what changes must be made for EDPs. The EDPs process and implementation of designed HBP need sustainable financial resources.

 The authors believe that enabling political environments and adequate and sustained financial resources are crucial determinants of the impact of EDPs for seeking UHC goals. Therefore, the EDPs should not be considered a short project and needs long-time efforts.

 Institutionalisation is not easy because any change in the setting priority process and extensive use of HTA has a redistributive characteristic. It affects the dynamic of financing and power. This is why the political economy approach is beneficial to analyse HBP conditions in countries.

 Moreover, when it shifts resources from one service to another, some stakeholders’ conflicts of interest will be provoked. So, good governance is needed during the design of HBP to ensure that the process follows elements of fairness. For this reason, the success of designing and implementing HBP and its institutionalisation is a critical issue that requires preparedness and the country’s readiness in addition to the technical aspects of the HBP design. In summary, this guide greatly supports countries in priority setting and designing HBP, focused on the EDPs in general conditions. For this reason, despite institutionalisation proposed for the success of HBP, it needed to be more explicit for sustainability towards UHC and increasing the potential impact of EDPs, if HBP considers as a broader policy change. In other words, since “fairness is the reasonableness of decisions perceived by stakeholders” and, in many low- and middle-income countries, setting the package is utilised as a part of a long-term policy change through UHC, institutionalisation should be considered as the fifth element of fairness of the EDPs besides the other four elements mentioned earlier. In this case, EDPs and HBP can contribute to a long-lasting initiative and sustainable health system strengthening towards UHC.

## Ethical issues

 Not applicable.

## Competing interests

 Authors declare that they have no competing interests.

## Authors’ contributions

 Conception and design: HSS, MJ, and RM; Drafting of the manuscript: HSS and RM; Critical revision of the manuscript for important intellectual content: HSS, MJ, and RM.
